# Steroid-refractory immune related hepatitis may hide viral re-activation

**DOI:** 10.2144/fsoa-2020-0056

**Published:** 2020-08-06

**Authors:** Fabrizio Citarella, Alessandro Galletti, Marco Russano, Paolo Gallo, Umberto Vespasiani-Gentilucci, Antonio Picardi, Giuseppe Tonini, Bruno Vincenzi, Daniele Santini

**Affiliations:** 1 Department of Medical Oncology, Campus Bio-Medico University, Via Alvaro del Portillo 200, Rome, Italy; 2 Department of Medicine, Unit of Internal Medicine & Hepatology, Campus Bio-Medico University, Via Alvaro del Portillo 200, Rome, Italy

**Keywords:** corticosteroids, hepatic immune-related toxicity, hepatitis B, immune-related adverse events, immunotherapy

## Abstract

Cancer immunotherapy has become a stronghold in modern oncology. Immune checkpoint inhibitors, in particular anti-PD-1 and anti-PD-L1 antibodies, are approved for the treatment of several solid cancers. In the near future, an increasing number of patients will be eligible for immunotherapy. Therefore, the management of immune-related adverse events is a daily challenge in clinical practice, among which hepatic immune-related toxicity has been described as a rare adverse event. We report the case of a patient treated with nivolumab (an anti-PD-L1 antibody) for a stage IV resected melanoma who developed recurrence of steroid-refractory liver toxicity that was later discovered to be associated with acute exacerbation of chronic undiagnosed hepatitis B. The patient significantly benefited from antiviral treatment. We conclude that serological viral screening is strongly recommended before starting immune checkpoint inhibitor treatment.

## Lay abstract

Transaminase elevation during treatment with immune checkpoint inhibitors may reveal the rare occurrence of a hepatic immune adverse event. Treatment of this side effect usually involves corticosteroids whose slow tapering is required in case of transaminases reduction. Corticosteroid-refractory hepatic impairment requires hepatic virus screening since immunotherapy may stimulate viral re-activation. We conclude here that early hepatic virus screening should be strongly recommended in order to identify concurrent viral replication when there is a hepatic immune adverse event.

## Clinical report

Hepatic toxicity during immunotherapy treatment usually presents as mild and asymptomatic elevations of liver enzymes but it can rarely be associated with serious events requiring discontinuation treatment and use of immunosuppressive agents [1]. Concomitant viral infections (hepatitis B and C virus, HIV) do not seem to influence toxicity and efficacy of immune checkpoint inhibitors [2] and only a few cases of hepatitis B activation are reported in literature [3–5]. European Society for Medical Oncology (ESMO) guidelines on management of toxicities from immunotherapy do not recommend a baseline hepatitis assessment [6].

**Figure 1.**
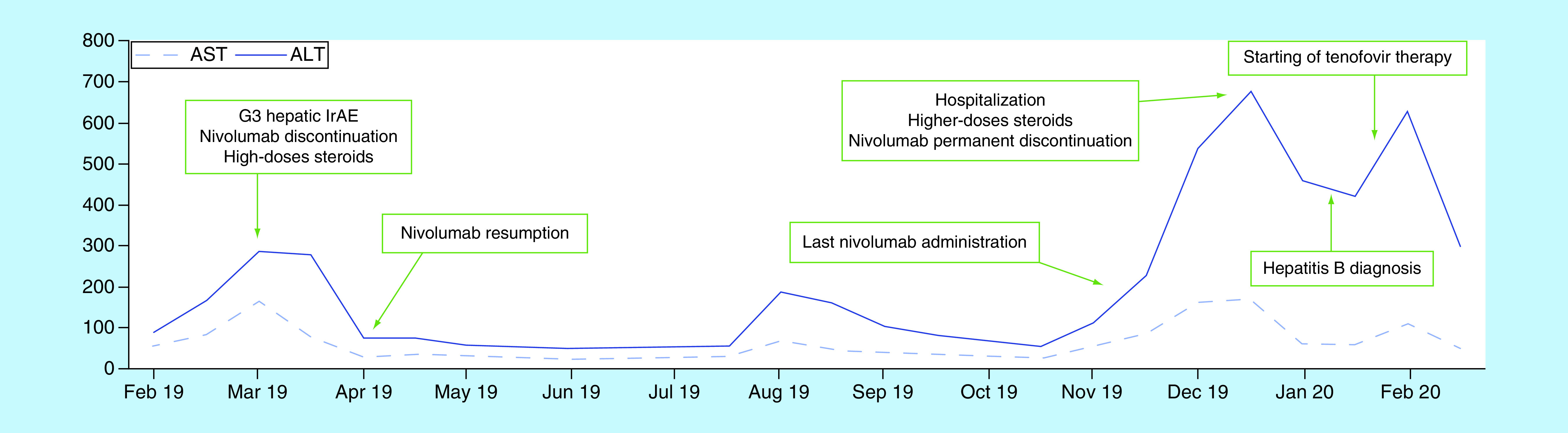
Timeline of transaminases levels upon immune checkpoint inhibitor treatment and hepatitis management. ULN: AST 34 U/l; ALT 55 U/l. ULN: Upper limit of normal.

We report the case of a 39-year-old Caucasian man who developed liver injury during treatment with adjuvant nivolumab for resected stage IV melanoma. The patient had neither comorbidities nor relevant baseline laboratory abnormalities with a slight long-lasting elevation of aminotransferases not otherwise diagnosed. Viral hepatitis screening was not performed. After the first administration of nivolumab (480 mg every 4 weeks flat-dosing schedule) the patient developed Grade 3 transaminases elevation according to CTCAE (Common Terminology Criteria for Adverse Events), without clinically relevant increase of bilirubin levels. After nivolumab discontinuation and high doses of systemic corticosteroids (intravenous methylprednisolone at starting dose of 2 mg/Kg followed by oral prednisone 1 mg/Kg once daily), liver enzymes returned to baseline values within two weeks. This first immune-related adverse events was not associated with symptoms or other laboratory abnormalities, so we decided to resume treatment with nivolumab (at fixed dose of 240 mg every 2 weeks) and closely monitor liver function. The patient received 12 doses of nivolumab without any adverse events and with stability of values of liver enzymes. Eight months after treatment resumption, G3 transaminases increase recurred. The patient had no jaundice but suffered from other common hepatitis-related manifestations such as fatigue, loss of appetite and flu-like symptoms. We decided to permanently discontinue nivolumab and the patient was hospitalized and treated with high doses (2 mg/Kg) of intravenous methylprednisolone. Ultrasound showed regular hepatic surface and did not detect liver metastases or other pathological findings while despite immunosuppressive treatment no improvement of liver enzyme was noted. Hepatologists suggested further tests aimed at excluding other potential causes of liver injury and hepatitis B infection was diagnosed. HBsAg (hepatitis B surface antigen) title was 25513 UI/ml, total antiHBc (hepatitis B core antibody) and antiHBe (hepatitis B E antigen) were positive, IgM antiHBc and HBe antigen were negative and hepatitis B virus-DNA was found elevated (and >170000000 UI/ml). An acute exacerbation of a chronic undiagnosed infection was supposed, resulting in prompt start of antiviral therapy with tenofovir disoproxil and slowly tapering the dose of steroid. To date, after 4 weeks of antiviral therapy, the patient has experienced complete clinical benefit and liver enzymes decrease. Figure 1 summarizes the hepatic impairment and the evolution of toxicity during immunotherapy treatment.

We suppose that the immunosuppressive doses of steroids used to treat immune hepatitis exacerbated pre-existing and unknown hepatitis B. The present case demonstrates that the well-timed evaluation of viral hepatitis status could optimize the management of potential immunotherapy-induced liver injury. For that reason, universal screening with serological tests for viral hepatitis B should be performed before starting treatment with immune checkpoint inhibitors.

## Future perspective

Prevalence of viral infections inducible by steroid-induced immunosuppression remains underrated. Clinical trials generally exclude patients with viral infections such as hepatitis B virus, hepatitis C virus and HIV. Both observational and prospective studies should include infected patients in order to better clarify if immunotherapy treatment exposes the individual to a higher risk of viral exacerbation. Basal screening for viral infection could optimize clinical management of immune-related toxicities.

**Executive summary**

**Current indications for patients treated with immune checkpoint inhibitors**

- Baseline viral screening is not routinely performed before treatment.
- Safety of immunotherapy in virus-infected patients is not properly estimated in prospective clinical trials.

**Clinical implications for virus-infected patients**

- Viral infections do not seem to correlate with higher risk of hepatotoxicity during immunotherapy.
- Viral screening is recommended for patients developing refractory hepatic impairment during immunotherapy.
- Since viral infections are still underestimated, prospective trials should include infected patients.

## References

[1] Jennings JJ, Mandaliya R, Nakshabandi A, Lewis JH Hepatotoxicity induced by immune checkpoint inhibitors: a comprehensive review including current and alternative management strategies. Expert Opin. Drug Metab. Toxicol. 15(3), 231–244 (2019). 30677306 10.1080/17425255.2019.1574744

[2] Shah NJ, Al-Shbool G, Blackburn M Safety and efficacy of immune checkpoint inhibitors (ICIs) in cancer patients with HIV, hepatitis B, or hepatitis C viral infection. J. Immunother. Cancer 7(1), 353 (2019). 31847881 10.1186/s40425-019-0771-1 PMC6918622

[3] Pandey A, Ezemenari S, Liaukovich M, Richard I, Boris A A rare case of pembrolizumab-induced reactivation of hepatitis B. Case Rep. Oncol. Med. ID 5985131 (2018). 10.1155/2018/5985131 PMC6207901 30416833

[4] Lake AC Hepatitis B reactivation in a long-term nonprogressor due to nivolumab therapy. AIDS 31(15), 2115–2118 (2017). 28906278 10.1097/QAD.0000000000001599

[5] Koksal AS, Toka B, Eminler AT, Hacibekiroglu I, Uslan MI, Parlak E HBV-related acute hepatitis due to immune checkpoint inhibitors in a patient with malignant melanoma. Ann. Oncol. 28(12), 3103–3104 (2017). 28945827 10.1093/annonc/mdx502

[6] Haanen JBAG, Carbonnel F, Robert C, ESMO Guidelines Committee Management of toxicities from immunotherapy: ESMO clinical practice guidelines for diagnosis, treatment and follow-up. Ann. Oncol. 29(Suppl. 4), iv264–iv266 (2018). 29917046 10.1093/annonc/mdy162

